# Transmission blocking activity of a standardized neem (*Azadirachta indica*) seed extract on the rodent malaria parasite *Plasmodium berghei *in its vector *Anopheles stephensi*

**DOI:** 10.1186/1475-2875-9-66

**Published:** 2010-03-02

**Authors:** Leonardo Lucantoni, Rakiswendé S Yerbanga, Giulio Lupidi, Luciano Pasqualini, Fulvio Esposito, Annette Habluetzel

**Affiliations:** 1 Scuola di Scienze del Farmaco e dei Prodotti della Salute, Università di Camerino, 62032 Camerino (MC), Italy

## Abstract

**Background:**

The wide use of gametocytocidal artemisinin-based combination therapy (ACT) lead to a reduction of *Plasmodium falciparum *transmission in several African endemic settings. An increased impact on malaria burden may be achieved through the development of improved transmission-blocking formulations, including molecules complementing the gametocytocidal effects of artemisinin derivatives and/or acting on *Plasmodium *stages developing in the vector. Azadirachtin, a limonoid (tetranortriterpenoid) abundant in neem (*Azadirachta indica*, Meliaceae) seeds, is a promising candidate, inhibiting *Plasmodium *exflagellation *in vitro *at low concentrations. This work aimed at assessing the transmission-blocking potential of NeemAzal^®^, an azadirachtin-enriched extract of neem seeds, using the rodent malaria *in vivo *model *Plasmodium berghei*/*Anopheles stephensi*.

**Methods:**

*Anopheles stephensi *females were offered a blood-meal on *P. berghei *infected, gametocytaemic BALB/c mice, treated intraperitoneally with NeemAzal, one hour before feeding. The transmission-blocking activity of the product was evaluated by assessing oocyst prevalence, oocyst density and capacity to infect healthy mice. To characterize the anti-plasmodial effects of NeemAzal^® ^on early midgut stages, i.e. zygotes and ookinetes, Giemsa-stained mosquito midgut smears were examined.

**Results:**

NeemAzal^® ^completely blocked *P. berghei *development in the vector, at an azadirachtin dose of 50 mg/kg mouse body weight. The totally 138 examined, treated mosquitoes (three experimental replications) did not reveal any oocyst and none of the healthy mice exposed to their bites developed parasitaemia. The examination of midgut content smears revealed a reduced number of zygotes and post-zygotic forms and the absence of mature ookinetes in treated mosquitoes. Post-zygotic forms showed several morphological alterations, compatible with the hypothesis of an azadirachtin interference with the functionality of the microtubule organizing centres and with the assembly of cytoskeletal microtubules, which are both fundamental processes in *Plasmodium *gametogenesis and ookinete formation.

**Conclusions:**

This work demonstrated *in vivo *transmission blocking activity of an azadirachtin-enriched neem seed extract at an azadirachtin dose compatible with 'druggability' requisites. These results and evidence of anti-plasmodial activity of neem products accumulated over the last years encourage to convey neem compounds into the drug discovery & development pipeline and to evaluate their potential for the design of novel or improved transmission-blocking remedies.

## Background

In the last decade, a renewed commitment to the fight against malaria has arisen from governments of endemic countries and international organizations, with the explicit aim to eliminate the disease in low transmission settings and to reduce its burden in high transmission areas through high coverage application of insecticide treated mosquito nets and artemisinin-based combination therapy (ACT) [1]. An important advantage of ACT is that, when applied on a wide scale, it can impact on transmission intensity of the disease, thanks to the gametocytocidal activity of artemisinin derivatives [2]. Artemether in particular significantly reduces the density of the *Plasmodium falciparum *sexual stages that persist in the circulation of patients treated with non-gametocytocidal drugs for more than three weeks [3]. The long gametocyte life span of *P. falciparum *allows the parasite to propagate to blood-seeking mosquitoes for a prolonged period, thus increasing its chances to be transmitted to other human hosts. Moreover, there is evidence that the stress imposed to parasites by drug treatment determines an up-regulation in the production of sexual stages [4]. These observations imply that treating patients with schizonticidal drugs has the unwanted detrimental 'side effect', from a public health perspective, of favouring the transmission of the parasite, thus jeopardising malaria control efforts at community level. Furthermore, this mechanism may enhance the spread of drug resistance, once resistant parasite clones have developed within a plasmodial population [4]. Conversely, it has been demonstrated that the use of ACT can counteract the spread of parasite strains already resistant to one of the partner drugs [5].

A number of clinical studies conducted over the last years to evaluate the curative efficacy of different formulations of ACT have evidenced the transmission-blocking potential of the treatments: artemisinin-, artemether- and artesunate-based combinations, unlike other drugs/drug combinations, such as chloroquine (CQ), quinine and sulphadoxine-pyrimethamine [6-8], were found to decrease the prevalence of gametocytaemia [9,10] and mosquito infection [11,12]. However, membrane feeding assays showed that around 60% and 40% of children treated with artemether-lumefantrine and sulphadoxine-pyrimethamine plus artesunate, respectively, were still infective to mosquitoes on day 14 after the beginning of the treatment [13], indicating that gametocytaemia reduction after ACT treatment is a slow process, requiring 3-4 weeks [14-16]. This represents an obvious limitation for the potential of ACT as a transmission-blocking tool. Also, the first evidences of *P. falciparum *resistance to artemisinin derivatives [17-19] are casting a shadow over the future of malaria control with ACT.

The search for new transmission-blocking compounds to back up the artemisinins' gametocytocidal action and to reduce the chances for the diffusion of resistant parasites is, therefore, of great importance. Such compounds ideally should be directed against the mature forms of gametocytes, and/or against the stages of the parasite developing in the vector.

Medicinal plants represent a possible source for the discovery of anti-plasmodial molecules. Extracts of the neem tree (*Azadirachta indica*, Meliaceae) are used traditionally to treat malaria and other illnesses in several endemic countries [20,21]. The efficacy of these extracts is attributed to limonoids, a class of highly oxygenated terpenoids [22], endowed with a range of biological properties including insecticidal, anti-microbial, anti-inflammatory and immuno-modulatory activities [23].

Several studies demonstrated that *A. indica *leaf, seed and stem bark extracts possess *in vitro *inhibitory activity on *P. falciparum *asexual stages [24-29]. *In vitro *screening of purified limonoids revealed that gedunin and nimbolide are the most active molecules against *P. falciparum *[24,30-32]. Gedunin was found to be effective against CQ-resistant strains of the parasite, with estimated IC_50 _of 0.72 and 1.25 in different studies [32,33].

*In vitro *gametocytocidal activity of neem leaf and seed extracts on *P. falciparum *has been reported [25,28]. In a recent study, in particular, a neem leaf extract was demonstrated to eliminate more than 90% of *P. falciparum *immature and mature gametocytes in culture, at a concentration of 2.5 μg/ml [29]. Azadirachtin, a limonoid present in neem leaves, and particularly abundant in neem seeds, was observed to inhibit *in vitro P. falciparum *and *P. berghei *microgamete exflagellation [34]. Cellular studies revealed that azadirachtin prevents the microgametocyte from completing the three endomitoses required for microgametogenesis, and inhibits cytoskeletal functions [35].

However, the *in vivo *inhibitory effect of azadirachtin on male gametogenesis, and the effect of azadirachtin and other limonoids on parasite stages developing in the vector have not been explored yet. This work aims at assessing the transmission blocking potential of NeemAzal^® ^(a standardized, azadirachtin enriched extract of neem seeds), using a rodent malaria model.

## Methods

### Experimental model

The rodent malaria model *P. berghei*/*An. stephensi*/BALB/c mouse was used for the experiments. This model has been validated as appropriate for obtaining inferences on the potential of compounds as *P. falciparum *transmission-blocking agents [36]. The *An. stephensi *colony was maintained at 30°C, >95% RH and a photoperiod of 12 h. BALB/c mice, used as vertebrate hosts, were reared in the animal facilities of the Department of Experimental Medicine and Public Health of the University of Camerino, Italy. Female mice weighing 16 - 19 g were used in the experiments. Blood meals were carried out on mice anaesthetized with xylazine : acepromazine 6.5 : 3.5, at a dosage of 11 mg/kg of the mixture. Experimental animal rearing and handling were fully compliant with the Italian Directive 116 of 10/27/92 on the "use and protection of laboratory animals", and in adherence with the European regulation (86/609) of 11/24/86 (licence no. 125/94A, issued by the Italian Ministry of Health).

The *P. berghei *ANKA (CQ-sensitive) strain was maintained by weekly blood transfer from infected to healthy mice. Every second month a cyclic passage was carried out by infecting mosquitoes through a blood meal on gametocytemic mice and, three weeks afterwards, by transferring the parasite to healthy mice through the bites of the sporozoite carrying mosquitoes. Mosquitoes infected with *P. berghei *were kept at 19 ± 1°C for the whole duration of the sporogonic cycle [37].

### Neem products

The following neem products were used: i) NeemAzal^® ^technical grade (NA; Trifolio-M GmbH, Lahnau, Germany), a standardized extract of *A. indica *seed kernels, containing limonoids at a concentration of 57.6% [38] and azadirachtin A at 34% (table 1); ii) azadirachtin tech grade (AZA; Sigma). Stock solutions of the neem products were prepared in absolute ethanol, at an azadirachtin A concentration of 50 mg/ml, according to manufacturers' information about azadirachtin content in the extracts.

**Table 1:** Limonoid content of NeemAzal^®^

azadirachtin A	34%
other azadirachtins (azadirachtin B to K)	16%
salannins	4%
nimbins	2%
**Total limonoids**	**56%**

Experimental mice received intraperitoneal (ip) inoculations of the various test solutions in a volume of 100 μl/mouse, obtained by dilution of the stock solutions with PBS (pH 6.5) with the addition of Tween 80 and DMSO, to final solvent concentrations of 5 - 20% ethanol, 7,5% Tween 80 and 10% DMSO. Control mice were inoculated with solutions containing solvents only, with 20% ethanol, the maximum alcoholic concentration present in the highest test dosage.

### Evaluation of the impact on the sporogonic development

Experimental mice were infected by ip inoculation of 10^7 ^infected red blood cells and, four days after infection, the presence of mature gametocytes was verified on Giemsa stained blood smears. Three gametocytemic mice were used for each of the five treatments, namely NA 13.2 mg/kg, NA 25 mg/kg, NA 50 mg/kg, AZA 50 mg/kg, and control solutions. Sixty minutes after receiving the treatment, the mice were narcotized and placed, according to the treatment, over cages containing ~250 *An. stephensi *females, 5 - 7 day old. Nearly all of the mosquitoes appeared successfully fed within 30 - 45 minutes. Unfed mosquitoes were discarded 24 hours after the blood meal. Ten days after the blood meal, samples of about 40 mosquito females per treatment group were dissected and their midguts examined under the light microscope (400×) to assess oocyst presence and count their number. Log transformed oocyst densities were compared using the Student's *t *test. Oocyst prevalences were compared using the χ^2 ^test. The experiment was replicated three times.

### Assessment of mosquito infectivity

As another measure to assess sporogonic cycle blockage by the neem treatments, healthy mice were challenged by 10 - 15 bites each, using the treated mosquitoes, three weeks after the infective blood meal. Two mice were used per treatment group and Giemsa-stained thin smears were observed to ascertain mouse positivity for *P. berghei *one week after the challenge.

### Evaluation of the impact on early sporogonic stages, developing in the midgut lumen

To determine the effect of neem substances on gamete-to-ookinete development mice were treated and exposed to mosquitoes as described above and mosquitoes from each treatment group were dissected to detect and count ookinetes in the midgut lumen, 18 and 20 hours after the blood meal, i.e. at a time when *P. berghei *gametogenesis, fecundation and ookinete development are completed. Midguts were excised in a drop of calf serum, transferred to Eppendorf tubes in pools of three and homogenized in 4 μl of calf serum. Three microliters of the homogenate were smeared onto a glass slide and stained with Giemsa. Ten to eleven slides (30-33 mosquitoes) were prepared for each treatment group. Zygotes, immature (retort stage to elongated shape with marginal nucleus) and mature ookinetes were counted over 300 microscopic fields (1,000×) on each slide, following a cross-shaped transect. The experiment was repeated twice. Log transformed midgut stages' densities were compared using the Student's *t *test.

To verify whether NA treatment altered the zygote and/or ookinete morphology and whether it inhibited the formation of mature ookinetes, 9 out of 21 slides from the two NA 50 mg/kg treatment replicates were extensively screened over a total area of 628 mm^2 ^(15% of total smear surface). Totally, 90 zygotes and ookinetes were found and inspected.

### Evaluation of the impact on oocyst maturation

**Figure 1:**
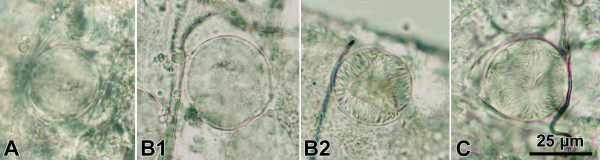
**Light microscope images (400×) of *Plasmodium berghei *oocyst development stages (day 10 after mosquito infection)**. Immature oocyst, with uniform content, **A**; immature oocysts, with sporoblasts and budding sporozoites, **B1 **and **B2**; mature oocyst, with fully developed sporozoites visible, **C**.

To establish if neem substances interfere with the maturation of oocysts, infected (untreated) mosquitoes were offered a second blood meal on neem-treated mice. About 1,800 *An. stephensi *females were infected with *P. berghei *through a blood meal on gametocytaemic mice. On day 7 after the infective blood meal, the mosquitoes were offered a second blood meal, according to the procedure described above, on three groups of three uninfected mice treated with 50 mg/kg NA, 50 mg/kg AZA, or control solutions. On day 10 after the first, infective blood meal (day 3 after the second blood meal), samples of mosquitoes from the three experimental groups were dissected, and oocyst numbers and their stage of development were recorded. The oocyst developmental status was coded into three categories, namely: s1, immature oocyst, before the formation of sporoblasts (Figure 1A); s2, immature oocyst, with visible sporoblasts and budding sporozoites (Figure 1B1 and 1B2); s3, mature oocyst containing fully developed sporozoites (Figure 1C). Log transformed oocyst densities were compared using the Student's *t *test.

## Results

### Block of the sporogonic development

The development of *P. berghei *in *An. stephensi *mosquitoes was blocked when the sexual stages of the parasite were ingested by females taking a blood meal on NA-treated, gametocytaemic mice. Within the NA 50 mg/kg treatment group, none of the 138 mosquitoes examined in three consecutive experiments revealed the presence of oocysts (Table 2).

**Table 2:** Effect of NeemAzal^® ^on *P. berghei *oocyst prevalence and density on mosquito midguts.

Treatment*	*Experiment*^**†**^	*% Prevalence (CI_***95***_)*	***Oocysts/mosquito (CI_***95***_)***^#^	*Examined mosquitoes*
control	1	90 (81 - 99)	17.5 (11.1 - 27.2)	40
	2	83 (68 - 99)	29.9 (15.8 - 55.8)	24
	3	100	111.2 (83.8 - 148.9)	40
	4	86 (72 - 100)	35.2 (21.6 - 57.6)	22
	5	88 (78 - 98)	11.3 (7.2 - 17.4)	42
	6	89 (80 - 97)	26.1 (15.8 - 42.4)	53
	*tot*^‡^	*90 (86 - 94)*	*27.7 (22.3 - 34.2)*	*221*

NA 13.2 mg/kg	4	82 (64 - 100)	49.9 (24.5 - 100.5)	17
	5	79 (66 - 91)	12.7 (8.6 - 18.7)	42
	6	100	39.4 (30.5 - 50.9)	58
	*tot*	*90 (84 - 95)*	*28.9 (23.0 - 36.3)*	*117*

NA 25 mg/kg	3	90 (81 - 99)	42.8 (26.7 - 69.1)	40
	5	69 (55 - 84)	8.9 (5.4 - 14.2)	39
	6	96 (91 - 100)	30.5 (22.3 - 41.1)	54
	*tot*	*86 (81 - 92)*	*25.6 (19.9 - 32.8)*	*133*

NA 50 mg/kg	1	0	0	55
	2	0	0	43
	3	0	0	40
	*tot*	*0*	*0*	*138*

AZA 50 mg/kg	3	95 (88 - 100)	23.3 (15.0 - 36.0)	41
	5	88 (78 - 98)	24.3 (17.2 - 34.5)	42
	6	83 (68 - 99)	3.2 (2.0 - 5.0)	24
	*tot*	*90 (73 - 100)*	*16.1 (12.2 - 21.2)*	*107*

Oocyst prevalence and intensity was assessed on day 10 after the infective blood meal carried out on mice treated with the indicated products and doses. * NA = NeemAzal^® ^technical grade (Trifolio-M GmbH); AZA = azadirachtin tech grade (Sigma). ^† ^each number corresponds to one experimentation involving different treatment groups. ^# ^geometric means, only positive mosquitoes included. ^‡ ^values refer to pooled data.

This blockage of the sporogonic development was confirmed by mouse challenge: all the healthy mice (n = 6) exposed to 10 - 15 bites of mosquitoes fed on the NA 50 mg/kg treated gametocytaemic mice failed to develop *Plasmodium *infections, whereas all the mice (n = 6) exposed to control mosquitoes had positive blood slides on day 7 after the challenge.

No impact on the sporogonic development could be detected in mosquitoes of the other neem treatment groups, namely NA 25 mg/kg, NA 13.2 mg/kg and AZA 50 mg/kg (Table 2). In these groups, the percentage of oocyst-positive mosquitoes was high in all occasions, most often above 80%, and very similar in treatment and control mosquitoes. Also, NA at these dosages and AZA did not appear to be able to reduce the number of oocysts and all the mice challenged with mosquitoes from these treatment groups developed parasitaemia.

### Impact on early sporogonic stages, developing in the midgut lumen

**Figure 2:**
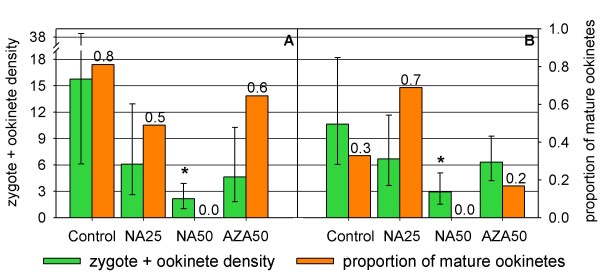
**Effects of the neem products on *P. berghei *midgut stages (zygotes to ookinetes)**. Parasites were counted on 300 microscopic fields (1000× magnification) of midgut content smears of *A. stephensi *females that had fed 18 (A), and 20 (B) hours before on mice treated with the indicated neem products and doses. NA25, NeemAzal^®^ 25 mg/kg; NA50, NeemAzal^®^ 50 mg/kg; AZA50, azadirachtin 50 mg/kg. Geometric means of zygote + ookinete densities, evaluated over ten smears (30 mosquitoes), with 95% confidence intervals (green bars; left axis). Mature ookinetes/total midgut forms ratio (orange bars; right axis). * means differ significantly from control (Student's *t *test; *p *≤ 0.002)

Mean early midgut stage (zygotes + ookinetes) densities were severely affected by NA action, as evidenced by mosquito midgut smears prepared 18 and 20 hours after the infective blood meal (Figure 2). Mean zygotes + ookinetes densities, estimated through counts on 300 microscopic fields (mf), amounted in the control mosquitoes 15.7 (CI_95 _6.1 - 38.4) and 10.6 (CI_95 _6.2 - 18.2) in the 1^st ^and 2^nd ^experiment, respectively, compared to only 2.1 (CI_95 _1.0 - 3.9) and 2.9 CI_95 _1.5 - 5.1) in mosquitoes fed on 50 mg/kg NA treated mice. These results show that NA at 50 mg/kg significantly reduced the number of parasites able to reach the zygote stage (*p *= 0.001 and *p *= 0.002 for the 1^st ^and 2^nd ^experiment, respectively).

The mean zygotes + ookinetes densities of mosquitoes fed on NA 25 mg/kg and AZA 50 mg/kg treated mice were lower than those of the controls, ranging from 4.6 to 6.7 (NA 25 mg/kg 1^st ^exp.: 6.1, CI_95 _2.6 - 12.9; 2^nd ^exp.: 6.7, CI_95 _3.7 - 11.7; AZA 50 mg/kg 1^st ^exp.: 4.6, CI_95 _1.8 - 10.3; 2^nd ^exp.: 6.3, CI_95 _4.2 - 9.2), but the difference was not statistically significant.

In both experimental groups, examined 18 or 20 hours after the infective blood meal, no mature ookinetes were detected on the midgut slides of NA 50 mg/kg treated mosquitoes (Figure 2), suggesting that the treatment interferes with the process of ookinete development. Accordingly, the additional extensive screening of 9 slides from this group, covering 15% of the total smear area, revealed the presence of 90 zygotes and post-zygotic forms, with no mature ookinete. For comparison, in control mosquitoes, 1/3 to 5/6 of the midgut stages present 18 or 20 hours after the infective blood meal were mature ookinetes (Figure 2).

**Figure 3:**
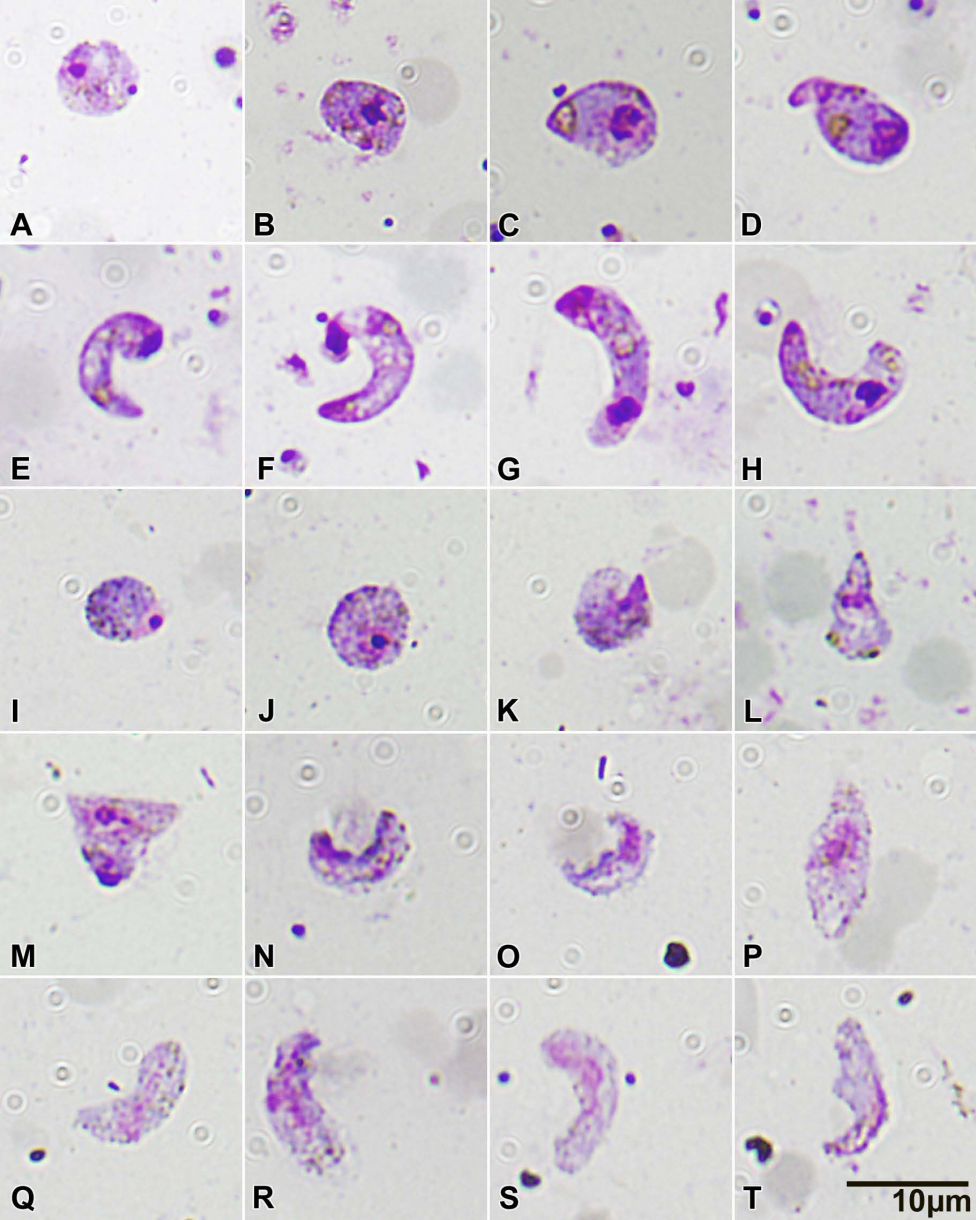
**Light microscope images (1000×) Giemsa stained *P. berghei *zygotes and ookinetes from *A. stephensi *midgut content smears, 18 - 20 hours after the infective blood meal**. Control mosquitoes, **A **to **H**; NeemAzal^® ^50 mg/kg treated mosquitoes, **I **to **T**. See text for details.

The morphological comparison of the zygotes and post-zygotic forms traced on NA 50 mg/kg slides with controls allowed alterations of the post-zygotic stages to be detected (Figure 3), whereas zygotes did not appear to differ. Giemsa-stained zygotes from both groups were similar in size, displaying rounded, condensed nuclei, intense cytoplasmic coloration and comparable haemozoin granules and crystalloid precursors (Figure 3A, 3I to 3J). In the control group, the ookinete development from retort stages (Figure 3B to 3F) to mature ookinetes (Figure 3G, 3H) was characterized by the completion of the elongation process, with the disappearance of the residual bulbous end containing the condensed nucleus, an intense staining of the apical region and the appearance of well defined haemozoin-surrounded crystalloid organelles. Several morphological alterations of the post-zygotic forms were found in the NA 50 mg/kg group (Figure 3K to 3T):

i) irregular cell shape (Figure 3L, 3M) and jagged cell borders (Figure 3K, 3N, 3O, 3R to 3T);

ii) lack of intense nuclear staining, with the nuclear material appearing dispersed within the cytoplasm (Figure 3O to 3T);

iii) weak staining, if any, of the apical region (Figure 3L, 3O to 3Q, 3S, 3T);

iv) lack of crystalloid assembly, the organelle granules remaining dispersed as in the zygote stage (Figure 3K, 3M to 3S), or partial crystalloid assembly (Figure 3L, 3T).

### Evaluation of impact on oocyst maturation

The exposure of early *P. berghei *oocysts to NA and AZA through a second treated blood meal on day 7 after mosquito infection did not affect the oocysts' subsequent development. In both neem treated groups oocysts underwent regular sporoblast formation and sporozoite maturation, as in untreated mosquitoes. Also, the proportion of mature oocysts containing fully developed sporozoites (stage s3) was similar in treated and control groups. Salivary glands were successfully invaded by sporozoites in both groups, indicating that neem treatment was not able to interfere with the sporogonic cycle (Table 3).

**Table 3:** *P. berghei *oocyst number/mosquito and development stage proportions after a secondary, treated blood meal.

*Treatment*	*Replication*	Stage of development*			*Total (CI95%)*	*Examined mosquitoes*
				
		*s1 (%)*	*s2 (%)*	*s3 (%)*		
control	1	25 (38)	23 (37)	12 (25)	70 (50 - 98)	27
	2	15 (33)	12 (31)	13 (36)	54 (42 - 70)	92

NA 50 mg/kg	1	10 (36)	10 (36)	8 (28)	31 (19 - 49)	23
	2	30 (42)	21 (33)	11 (25)	82 (68 - 100)	112

AZA 50 mg/kg	1	19 (34)	20 (35)	18 (31)	61 (40 - 93)	27

Secondary blood meals were carried out on mice treated with the indicated products and doses on day 7 after mosquito infection. Oocysts were evaluated on day 3 after the secondary blood meal (day 10 post infection). *s1: immature oocysts, before the formation of sporoblasts (fig. 1A); s2: immature oocysts, with visible sporoblasts and budding sporozoites (fig. 1 B1 - B2); s3: mature oocysts containing fully developed sporozoites (fig. 1C). Geometric means, only positive mosquitoes included.

## Discussion

NeemAzal^® ^(NA), a commercial *A. indica *seed extract, containing the limonoid azadirachtin as the main component, completely blocked the development of *P. berghei *in *An. stephensi *mosquitoes fed on gametocytaemic mice treated with the extract at 50 mg/kg body weight. The treated mosquitoes did not produce oocysts and were thus unable to infect healthy mice. The examination of slides prepared from the midgut content of experimental mosquitoes showed that NA activity was directed to the early sporogonic stages: the total number of zygotes and post-zygotic forms was reduced in the NA-treated group compared to controls, indicating that NA components interfere with parasite development already before zygote formation. In addition, post-zygotic forms from NA treated mosquitoes displayed evident morphological alterations and no mature ookinetes could be detected. These results indicate that NA may act on gametogenesis and ookinete formation.

*In vitro *studies conducted by Jones and colleagues [34] showed that azadirachtin interferes with the exflagellation process of *P. falciparum *and *P. berghei *microgametocytes, inducing an interruption of the endomitotic divisions and the formation of rigid extensions on axonemes, preventing their motility [34]. In a subsequent study it was demonstrated by Billker *et al *[35] that azadirachtin disrupts cytoplasmic and axonemal microtubule organization, possibly by compromising the functionality of the microtubule organizing centres (MTOC), including spindle plaques. The results presented in this report suggest that azadirachtin inhibits microgametogenesis *in vivo*.

The morphological alterations observed on NA-treated post-zygotic forms are compatible with the hypothesis that azadirachtin interferes with ookinete formation via a possibly similar, microtubule-targeted mechanism of action. The irregular cell shape, the jagged cell membrane, the inability to form the typically strongly stained apical complex and to perform the elongation process are compatible with an interference with the microtubule organizing processes involved in cytoskeletal and organelle rearrangements. Electron microscopy studies have shown that ookinete development is depending on the differentiation of a MTOC at the apical pole, from which subpellicular, longitudinal microtubules radiate and the triple membrane (pellicle) is formed [39,40]. Further evidence for azadirachtin action on microtubule organizing processes is available from studies of Salehzadeh and colleagues [41], who showed that the antimitotic activity of azadirachtin observed on cultured insect cells was related to its interference with tubulin polymerization, through a mode of action resembling that of colchicine. Colchicine and other compounds of natural origin, added to zygote cultures of *Plasmodium gallinaceum*, have been demonstrated to inhibit ookinete formation [42].

The fact that a transmission blocking action was observed with NA, but not with AZA tested at the same dose, might suggest that other bioactive limonoids present in the former (which is an azadirachtin-enriched extract, not a pure azadirachtin molecule) could explain the difference in activity. However, a HPLC analysis conducted according to the protocol of Kaushik [43] did not evidence additional compounds present in significant amounts in NA, but revealed that the azadirachtin A content of AZA was about half the amount declared by the manufacturer, which could well explain the difference in transmission blocking effect observed with the two products.

Numerous studies demonstrated that azadirachtin and other limonoids present in neem extracts are active on insects [44], including malaria vectors [45-47]. Exploring the insecticidal properties of NA in *An. stephensi*, it was found that mosquitoes fed with artificial blood meals or sugar solutions containing a NA concentration corresponding to 100 μg/ml azadirachtin displayed reduced blood feeding capability and produced fewer eggs. This reduction in oviposition was mirrored by a degenerative tissue damage provoked by NA on ovarian follicles [48].

These multiple and diverse effects on *Plasmodium *midgut stages and female mosquitoes are of remarkable interest in view of the development of transmission blocking molecular tools, directed against both the malaria parasite and the malaria vector, based on neem extracts. To challenge their potential, the identification of the bioactive molecules and the characterization with respect to target stage and mode of action represent important research questions to be addressed. Also, the 'druggability' of limonoids may represent a major challenge: the few studies that explored limonoid pharmacokinetics indicate a relatively short half-life and low bioavailability of the compounds. Manners *et al *[49] report that limonin and related metabolites were not detectable in plasma samples of human volunteers 24 hours after the administration of 30 mg/kg of pure limonin glucoside, which reached its peak plasma concentration 6 hours post-administration. Lack of *in vivo *curative activity of gedunin against *P. berghei *has been ascribed to its poor absorption in the digestive tract [20].

Developing transmission blocking drug candidates from neem compounds is a long and challenging way to go. Still, evidences accumulated over the years on the anti-plasmodial and mosquito-toxic activity of plants encourage medicinal chemistry studies aiming to develop limonoid leads with improved bioavailability characteristics. Once limonoid leads are obtained, drug interaction studies with ACT would be required and possible interactions with the immune system should be assessed, before considering them for further development as transmission-blocking components of combination therapies.

As an equally challenging alternative, the development of neem-based transmission blocking remedies by improving traditional herbal treatments could be explored. Extract fractions displaying a high activity against gametocytes and/or against the *Plasmodium *stages developing in the vector can be identified applying bio-guided fractionation methods. Combining the most active fractions should result in remedies with an increased overall transmission blocking efficacy, thanks to potentially additive or synergistic effects of azadirachtin, other limonoids and other components present in extracts [50]. In addition the presence of various bioactive molecules in the extracts might reduce the likelihood of selecting resistant strains in the target organisms [45]. On the other hand, toxicity and variability of preparations must be taken into account when considering plant extracts for therapeutic use, as the useful metabolites' concentration may vary widely according to environmental conditions, geographic origin, mode of plant collection and storage [51]. In the case of neem preparations, not only toxicity risks, but possible immuno-modulatory effects also need to be explored before considering a product for human use [52].

## Conclusion

The neem tree, *A. indica*, produces and stores in its organs and tissues secondary metabolites that have been demonstrated to be active against different malaria parasite stages in the vertebrate host and to possess insecticidal and insect growth regulatory properties on malaria vectors as well. The transmission blocking activity of a standardized, azadirachtin rich neem extract described in this work represents an additional element contributing to the potential of neem as a resource for malaria control. The available evidence strongly encourages to convey neem compounds in the drug discovery and development pipeline and also to evaluate the potential of plant extracts for the development of improved traditional remedies.

## Competing interests

The authors declare that they have no competing interests.

## Authors' contributions

LL participated in study design, carried out the *in vivo *experiments, performed the statistical analysis, and drafted the manuscript; RSY participated in study design and in the execution of the experiments; GL carried out the HPLC analysis and helped with study design; LP participated in the execution of the *in vivo *experiments; FE participated in study design and helped with manuscript revision; AH coordinated the work, participated in study design and critically revised the manuscript. All the authors read and approved the final manuscript.
